# A touch screen-automated cognitive test battery reveals impaired attention, memory abnormalities, and increased response inhibition in the TgCRND8 mouse model of Alzheimer's disease

**DOI:** 10.1016/j.neurobiolaging.2012.08.006

**Published:** 2013-03

**Authors:** Carola Romberg, Alexa E. Horner, Timothy J. Bussey, Lisa M. Saksida

**Affiliations:** a Department of Experimental Psychology, University of Cambridge, Cambridge, UK; b Wellcome Trust and MRC Behavioural and Clinical Neuroscience Institute, University of Cambridge, Cambridge, UK

**Keywords:** Alzheimer's disease, Mouse models, Attention, Response control, Touchscreen, Memory, Cognition, β-amyloid, TgCRND8

## Abstract

Transgenic mouse models of Alzheimer's disease (AD) with abundant β-amyloid develop memory impairments. However, multiple nonmnemonic cognitive domains such as attention and executive control are also compromised early in AD individuals, but have not been routinely assessed in animal models. Here, we assessed the cognitive abilities of TgCRND8 mice—a widely used model of β-amyloid pathology—with a touch screen-based automated test battery. The test battery comprises highly translatable tests of multiple cognitive constructs impaired in human AD, such as memory, attention, and response control, as well as appropriate control tasks. We found that familial AD mutations affect not only memory, but also cause significant alterations of sustained attention and behavioral flexibility. Because changes in attention and response inhibition may affect performance on tests of other cognitive abilities including memory, our findings have important consequences for the assessment of disease mechanisms and therapeutics in animal models of AD. A more comprehensive phenotyping with specialized, multicomponent cognitive test batteries for mice might significantly advance translation from preclinical mouse studies to the clinic.

## 1 Introduction

Alzheimer's disease (AD) is a progressive, neurodegenerative disease that is characterized by an accelerated decline of cognitive abilities. Because plaques of aggregated β-amyloid (Aβ) peptide and tangles of hyperphosphorylated tau protein are prominent anatomical hallmarks in the brains of AD patients (Braak and Braak, 1991, 1995), the discovery of mutations in genes encoding these peptides in familial AD patients promised to be a major breakthrough for the understanding of the disease. Novel transgenic mice carrying these pathogenic human genes provided the possibility of addressing how altered amyloid precursor protein (APP) or tau processing may affect the functional integrity of the underlying neuronal networks (Götz and Ittner, 2008; Sofroniew and Staley, 1991). Many studies have since used such mice to implicate altered APP in the development of learning and memory deficits (Eriksen and Janus, 2007). Moreover, these memory deficits have been remedied by various treatments aimed at reducing Aβ load (Zahs and Ashe, 2010). However, the translation of these findings into successful treatments for AD patients has brought disappointing results. One reason for this might be the limited translatability of the rodent tests. Indeed, conventional rodent testing procedures (e.g., water maze, Y-maze) have very different procedural and cognitive demands to the kinds of computer-automated cognitive test batteries increasingly used for the diagnosis of patients. A related issue is that animal studies have focused almost exclusively on memory. While memory impairment is one of the most prominent features of early AD, multiple cognitive domains are compromised in the majority of AD individuals: deficits of attention and executive functioning (inhibitory control) occur early in the disease and can precede language and spatial impairments (Bentley et al., 2008; Grady et al., 1988; Perry and Hodges, 1999; Sahakian et al., 1989). Indeed, the current criteria for the diagnosis of probable AD stipulate deterioration of 2 or more areas of cognition of sufficient magnitude to interfere with work or social function (McKhann et al., 1984, 2011; Waldemar et al., 2007).

Thus, a more complete cognitive assessment may go some way toward improving translation from mouse to human, especially because apparent memory deficits can actually be secondary to deficits in attention, motivation, or response control. Therefore, we tested an exemplary mouse model of AD-typical amyloid pathology—the widely used TgCRND8 mouse (Chishti et al., 2001)—on a cognitive test battery assessing multiple cognitive domains. To overcome strain-specific physiological anomalies and behavioral limitations (e.g., poor vision in C3H background strains), we used TgCRND8 mice on a hybrid C57Bl/6:129Sv background, with well-characterized Aβ pathology (Adalbert et al., 2009). By taking advantage of novel touch screen technology (Bussey et al., 2001, 2012; Morton et al., 2006; Romberg et al., 2011) we employed a set of tasks that are procedurally similar to neuropsychological tasks used in humans. We tested mice on a battery of 5 tasks designed to assess a range of cognitive abilities thought to be affected in AD, along with an appropriate control task assessing cognitive domains largely spared in AD patients (Table 1) and control measures for gross behavioral abnormalities, such as altered motivation or activity levels. Importantly, all tasks are computer-automated and carried out in the same apparatus using the same types of stimuli, responses, and reinforcers, thus minimizing the likelihood of experimenter bias and minimizing confounds when comparing data across different tests.

## 2 Methods

### 2.1 Animals

Specific mutations can have different phenotypes in different genetic backgrounds (Chishti et al., 2001; Gerlai, 1996). In order to control for background-specific genetic elements potentially modifying the degree of pathology and/or behavior, we employed TgCRND8 mice on a hybrid C57Bl/6:129Sv background, 2 inbred mouse strains particularly suited to and widely used for behavioral testing. Unlike the original TgCRND8 mice (Chishti et al., 2001), these mice contain no genetic material from the C3H strain, which is unsuitable for most cognitive tasks due to visual impairment (Wong and Brown, 2006).

Hemizygous male TgCRND8 mice on a 50:50 C57BL/6:129Sv background (Adalbert et al., 2010; Chishti et al., 2001) were received from the laboratory of Michael Coleman at the Babraham Institute, Cambridge, UK. Testing cohorts of male TgCRND8 and wild type littermates were bred on site by mating TgCRND8 mice with wild type females of the same genetic background. The offspring were genotyped by polymerase chain reaction (primers: 5′-GCCTTTGAATTGAGTCCATCACG-3′; 5′-AGAAACGCCAAGCGCCGTGACT-3′). Mice were 4–5 months old at the onset of behavioral testing, at which stage they show profound Aβ-plaque pathology in the cortex and hippocampus (Adalbert et al., 2009; Chishti et al., 2001). To ensure a comparable level of disease progression, testing was performed with 3 cohorts: cohort 1 was used for the object recognition task (experiment 1), cohort 2 was tested on visual discrimination, reversal, and extinction (experiment 2), and cohort 3 was tested on the 5-choice serial reaction time task (5-CSRTT) (experiment 3), which was immediately followed by the extinction paradigm (experiment 4).

Animals were housed in groups of 1–4 mice, in a room with a 12-hour light/dark cycle (lights off at 7:00 pm). All behavioral testing was conducted during the light phase of the cycle. Mice were maintained on a restricted diet and kept at 85% of free-feeding body weight during behavioral testing. Water was available ad libitum throughout the experiment. All experimentation was conducted in accordance with the United Kingdom Animals (Scientific Procedures) Act (1986).

### 2.2 Apparatus

Testing was conducted in a custom-built, touch screen-based automated operant system for mice. The apparatus consisted of a standard modular testing chamber housed within a sound- and light-attenuating box (40 × 34 × 42 cm, Med Associates, Inc., St. Albans, VT, USA). The sound-attenuating box was fitted with a fan (for ventilation and masking of extraneous noise) and a 14 mg pellet dispenser. The operant chamber contained a 3 W house light, a tone generator, and a pellet receptacle (magazine, attached to pellet dispenser), which was illuminated by a 3 W light bulb and fitted with a photocell head entry detector. At the end of the box, opposite the magazine was a flat-screen monitor equipped with an infrared touch screen (16 cm high and 21.20 cm wide, Craft Data, Limited, Chesham, UK) mediated by ELO touch screen software V. 5.4 (ELO Touchsystems, Inc., Menlo Park, CA, USA). A black Perspex “mask” with task-specific response windows was placed over the screen, to reduce unintended responding by tail or other body parts. Stimulus delivery/detection and operant box input/outputs were controlled with custom written software (“MouseCat”, L.M. Saksida) written in Visual Basic 6 (Microsoft, Redmond, WA, USA).

Spontaneous object recognition was assessed in similar, now commercially available touch screen chambers from Campden Instruments, Loughborough, UK (Bussey-Saksida touch screen chamber for mice) and the associated software (AbetII, Lafayette Instruments, Lafayette, IN, USA). Although in general comparable with the touch screen boxes used for all other tasks, these chambers have a triangular shape and allow the placement of the reward tray at the front, separating the touch screen in 2 halves. The presence of infrared beams at the rear and in front of each half of the touch screen enable the recording of motor activity levels and mouse position.

### 2.3 Automated, spontaneous, object recognition task

This task is analogous to classic spontaneous recognition tasks run in the open field or Y-maze (Bartko et al., 2007), and involves neither food reward nor pretraining, because only spontaneous behavior is measured. Instead of real objects, pictures of objects are displayed on the touch screen, and exploration is measured as the number of touches to each stimulus, as well as the number of approaches toward each stimulus (i.e., infrared beam breaks in front of the respective image). Initially, animals were habituated to the touch screen chambers for 15 minutes per day, for 2 days in a row (house light off, touch screen blank). After habituation, each animal received 2 test sessions separated by at least 24 hours. Each test session started with a sample phase, followed by a 1-minute or 1-hour delay, and finished with a choice phase. At the beginning of the sample phase, the mouse was placed in the touch screen box (touch screen blank, house light off). When the animal triggered the photo beam at the back of the chamber, 2 identical objects were displayed, 1 in each of the 2 response windows on either side of the touch screen, and remained lit for 30 seconds. During this period, the number of touches of each object and the number of approaches toward each object (break of infrared beam in front of the stimulus) was recorded. After a variable interval between 20 and 50 seconds, where the screen remained blank and inactive, a new trial started and the same stimuli were displayed again as soon as the animal triggered the rear photo beam. After 10 trials or a maximum of 20 minutes, mice were either returned to their home cage for 1 hour before the choice phase, or the choice phase started immediately (after a 1-minute delay). The choice phase was identical to the sample phase, except that the object pair consisted of the familiar, previously encountered object and 1 brightness-matched, novel object. Object order (i.e., whether object A or B of a given set served as sample) as well as the presentation side of the novel object (i.e., left or right screen) was counterbalanced across animals of each group. Object pairs were matched for brightness (63% of the surface was black, 37% of the surface was white), and had previously been tested for biases (Bussey et al., 2001, 2008; Morton et al., 2006). The selection of stimuli (approximately 8 × 8 cm) was based on a visual acuity of 0.3 cycles per degree for C57Bl/6 and 129-Sv mice (Wong and Brown, 2006), which roughly corresponds to the ability to separate 2 lines spaced 1 cm apart from a distance of 20 cm. A preference score was calculated by dividing the difference between touches/approaches to the novel object and touches/approaches to the familiar object by the sum of touches/approaches to the novel object and touches/approaches to the familiar object. To ensure comparable exploration of the sample object, and a reliable measure of preference during the choice phase, mice were required to complete all 10 trials of the sample phase, and 8 trials of the choice phase, and to make more than 10/8 touches (to either object) in the sample/choice phases, respectively. Trials on which a mouse failed to meet these criteria were excluded from the analysis. The same criteria were used for the analysis of approaches.

### 2.4 Shaping for reward-based touch screen tasks

Mice were trained to operate the touch screen by a series of shaping procedures. For the first 2 days, animals were habituated to the operant box environment for 15 minutes a day. During this phase, the house light was on, the touch screen remained inactive, and the magazine was filled with 10 reward pellets. The following Pavlovian phase (1 day, 30 trials) differed between tasks. For animals proceeding on the visual discrimination tasks (cohort 2), 1 of 20 randomly shaped, black and white stimuli (approximately 7 × 7 cm) was randomly displayed in 1 of 2 response windows. For animals continuing on the 5-CSRTT (cohort 3), a white square (2 cm × 2 cm) appeared in 1 of 5 response windows. After 30 seconds, the stimulus disappeared, coinciding with a tone, the onset of the magazine light, and the delivery of a reward pellet. When the animal collected the reward, the magazine light extinguished and the next trial commenced with the delivery of a new stimulus. A response to the stimulus on the screen was rewarded with the delivery of a tone and 3 extra pellets. At the next stage, the stimulus remained on the screen until the mouse touched the stimulus, which was rewarded with a single pellet, tone, and illumination of the magazine light. The collection of the reward pellet triggered a 5-second intertrial interval (ITI; house light on, no stimulus, magazine inactive) after which the next trial commenced, and a new stimulus was presented in 1 of the 2/5 windows. Training continued until the animal completed 30 trials within 15 minutes for 2 consecutive days in a row. The final stage of shaping introduced the initiation procedure. At the onset of each trial, the magazine was illuminated, and the animal was required to initiate the stimulus delivery by a nose poke into the magazine. Successful initiation was indicated by the extinction of the magazine light and the subsequent display of a stimulus. After touching the stimulus and collecting the reward, a 5-second ITI was implemented (house light on, no stimulus), before the onset of the magazine light indicated the beginning of the new trial. When animals readily initiated trials and completed 30 trials within 20 minutes for 2 consecutive days, they were moved on to one of the following tasks.

### 2.5 Visual discrimination, reversal, and retention

#### 2.5.1 Acquisition

Mice were presented with a pair of black and white, brightness-matched stimuli on the touch screen, one of which was correct (S+) and the other incorrect (S−) (Bartko et al., 2011; same features as stimuli used for spontaneous object recognition, see Section 2.3.). A nose poke to the S+ resulted in a tone, magazine light, and a reward pellet. Incorrect responses were followed by a 5-second time-out in which the house light was extinguished, followed by a correction procedure in which the stimulus display was repeated until the mouse made a correct response (correction trial). Each daily session consisted of 30 trials, separated by an ITI of 20 seconds. Performance was measured by calculating the percentage correct choices per session of 30 (noncorrection) trials. When an individual animal reached more than 80% correct choices for 2 days in a row, acquisition was paused.

#### 2.5.2 Reversal

When all animals in a cohort had reached the acquisition criterion, all mice were put back on the task and received 3 further task sessions to reinforce reward contingencies and ensure stable baseline performance. The following day, contingencies of S+ and S− were reversed, i.e., the former S− was now correct and rewarded, and the former S+ incorrect and punished with a 5-second time-out. Because mice needed a large number of correction trials after the reversal, they only received 10 trials per session for the first 3 sessions. Data from these 3 sessions were collapsed into 1 data point. The reversal phase was stopped after 10 sessions, but not before the group performance reached more than 80% correct choices for 3 days in a row.

#### 2.5.3 Retention

To test that memory of the reward contingencies was stable and lasting, all mice received 2 further sessions with the same reward contingencies 10 days after the end of the last reversal day.

### 2.6 Touch screen 5-CSRTT

#### 2.6.1 Training phase

The general 5-CSRTT task procedure in the touch screen was described previously (Romberg et al., 2011). Mice were trained to respond to brief flashes of light pseudorandomly displayed in 1 of the 5 spatial locations on the touch screen. Mice were tested 5–6 days a week, 50 trials a day (or up to 1 hour). Each trial commenced with the illumination of the magazine light. In contrast to the last stage of shaping, a nose poke to the magazine did not result in the immediate display of a stimulus. Instead, the stimulus was delivered after a 5-second delay (the delay period), during which the animal was required to attend to the screen. If an animal prematurely touched the screen during this delay, the response was recorded as premature and the mouse was punished with a 5-second time-out (house light off, magazine inactive). The time-out was followed by a 5-second ITI (house light on, magazine inactive), after which the illumination of the magazine light signaled the onset of the next trial. The stimulus duration was initially set to 4 seconds, followed by a limited holding period of 5 seconds, during which the stimulus was absent but the animal was still able to respond to the location (limited holding period). Responses during stimulus presence or limited holding period were recorded either as correct (response to the stimulus window) or incorrect (response to any other window). A correct choice was rewarded with a tone, and pellet delivery, indicated by the illumination of the magazine light. Reward collection turned the magazine light off and triggered an ITI of 5 seconds. An incorrect response was punished with a 5-second time-out, followed by a 5-second ITI. A failure to respond to any window either during stimulus display or limited holding period was recorded as an omission and punished with a 5-second time-out, followed by a 5-second ITI. Additional, perseverative responses to the screen after premature (during time-out), correct (before collecting the reward), and incorrect (during time-out) choices were also recorded.

When the performance of a mouse stabilized at 4-second stimulus duration (> 80% accuracy, < 20% omissions on 3 out of 4 consecutive days), the stimulus duration was reduced to 2 seconds. After reaching criterion with the 2-second stimulus, animals were tested for 2 more days. The mean measures of those 2 days were used to analyze baseline performance.

#### 2.6.2 Probe trials

After completing training at 2-second stimulus duration, animals were challenged with an increased attentional demand by reducing the stimulus duration to 1.5 seconds, 1 second, 0.8 seconds, and 0.6 seconds. To control for order effects, the sequence of stimulus durations presented to each animal in a group was randomized in a Latin square design. Each animal performed 2 consecutive days at a given stimulus duration, and was then moved back onto a 2-second stimulus duration for 2 days, or until it re-reached criterion (> 80% accuracy, < 20% omissions).

Attention and response control were assessed by measuring response accuracy (correct trials divided by correct plus incorrect trials in %), omissions (omitted trials divided by total trials in %), premature responses (premature trials divided by total trials), perseverative responses (perseverative responses per choice), and response and magazine latencies after correct choices (in ms).

### 2.7 Extinction

#### 2.7.1 Training

After completion of the 5-CSRTT, mice underwent an extinction protocol. Upon trial initiation, a single stimulus was presented in the middle window of the 5-hole mask.

A nose poke to the illuminated window was rewarded, whereas a nose poke to any other window was followed by a time-out. Each daily session consisted of 30 trials (10-second ITI). Training continued until an animal achieved a choice accuracy of > 95% for 5 days in a row.

#### 2.7.2 Extinction

During the extinction phase, the stimulus was again displayed in the middle window, although no initiation was required. The stimulus disappeared when the animal touched the window or after 10 seconds, but no tone or reward was delivered. Each session comprised 30 trials. Performance was expressed as % responses to the stimulus per session.

### 2.8 Data analysis

Means are displayed with standard errors and were submitted to 1-way or repeated measures (RM) analysis of variance (ANOVA) as indicated. Simple main effects were used for post hoc analyses of within-subject effects. All statistical analyses were conducted with a significance level of α = 0.05, using SPSS version 17 (SPSS Inc., IBM Corp., Armonk, NY, USA).

## 3 Results

### 3.1 Body weights

As described previously (Adalbert et al., 2009), the appearance of TgCRND8 mice and wild type mice was indistinguishable. Mean free feeding weights of all animals used in this study at the age of 4–5 months were similar between genotypes (TgCRND8: 28.2 g ± 0.9, *n* = 26; wild type: 29.2 ± 0.8 g, *n* = 26; Student *t* test, *t* < 1, *p* > 0.05).

### 3.2 Experiment 1: automated spontaneous object recognition

One of the first cognitive domains deteriorating in AD is explicit/declarative memory. A widely used memory task thought to assess similar memory processes in rodents is the objection recognition task run in the open field or Y-maze (Bartko et al., 2007). The task is motivated by an animals' inherent tendency to explore novel objects, and measures recognition memory by the degree of exploration of novel compared with familiar objects. In the conventional version of the task, rodents explore real 3-D objects, and exploration is measured manually according to more or less well-defined behavioral criteria. Because manual scoring of exploration is notoriously sensitive to experimenter bias, we have developed an automated, touch screen-based version of the object recognition paradigm, where animals explore 2-dimensional images of objects displayed on the touch screen. Object exploration is measured as the number of touches to each image, and as the number of times an animal approaches an object (infrared beam breaks in front of each image). Like the classic version of the object recognition task, and unlike all other tasks in this touch screen battery, this task does not involve a food reward.

The testing cohort (cohort 1) consisted of 5-month-old wild type (*n* = 9) and TgCRND8 (*n* = 11) mice. During the sample phase, 2 copies of a sample object were repeatedly displayed on the touch screen. After a delay (either 1 minute or 1 hour), the animal was presented with 1 copy of the sample object and a novel object. The degree of exploration of the novel object relative to the familiar object is taken as a measure of memory for the sample object (discrimination ratio).

Wild type and TgCRND8 mice explored the sample objects to a similar extent. Both the number of touches as well as the number of approaches toward the sample objects on the screen were similar between genotypes (touches: wild type 36.8 ± 3.6, TgCRND8 41.7 ± 1.7; *t* = 1.04, *p* > 0.1; approaches: wild type 32.1 ± 2.3, TgCRND8 39.6 ± 3.4; *t* = 1.7, *p* > 0.1), suggesting that the general motivation to explore novel objects is unaltered in TgCRND8 mice. Moreover, the number of infrared beam breaks (front and rear) during the entire sample phase (20 minutes), a measure of overall motor activity, did not differ between genotypes (mean of 4 sample phases; wild type: 255.6 ± 18.9; TgCRND8: 245.8 ± 30.2; *t* < 1, *p* > 0.3).

**Fig. 1:**
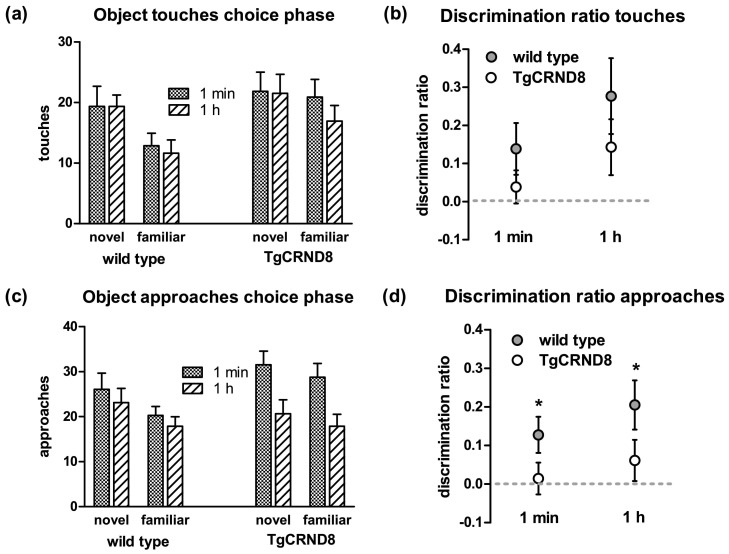
Automated spontaneous object recognition. (a) Mean total touches to novel and familiar object during the choice phase for each genotype and delay. (b) Discrimination ratio based on object touches (difference between novel object touches and familiar object touches divided by total object exploration (novel object touches − familiar object touches)/(novel object touches + familiar object touches)). Thus, a score of 0 corresponds to no preference for the novel object, whereas a score of 1 corresponds to exploration of the novel object only. (c) Mean total approaches to novel and familiar object during the choice phase for each genotype and delay. (d) Discrimination ratio based on object approaches. The discrimination ratio was calculated from the total number of approaches as in (b): ((novel object approaches − familiar object approaches)/(novel object approaches + familiar object approaches)). Data are presented as mean ± standard error of the mean. * *p* < 0.05.

On the choice phase, wild type mice explored the novel image more than the familiar image, regardless of whether the delay after the sample phase was 1 minute or 1 hour (Fig.). In contrast, TgCRND8 mice appeared to have no preference for either image (Fig.). These findings were reflected in the discrimination ratios, i.e., the relative exploration of novel versus familiar image: wild type mice showed greater discrimination than TgCRND8 mice with both delays, although there was only a trend of a genotype effect (Fig. 1b, RM ANOVA with genotype as between subjects factor and delay as within subject factor, main effect of genotype: *F*(1,18) = 2.7, *p* = 0.07, no main effect of delay or interactions).

A similar pattern of results was observed when object exploration was measured as the number of approaches toward an object (Fig. 1c and d). Wild type mice made more approaches toward the novel object (Fig. 1c) whereas TgCRND8 mice approached both objects similarly often (Fig. 1c). Consequently, wild type mice showed significantly greater discrimination ratios than TgCRND8 mice, irrespective of the duration of the delay (Fig. 1d, RM ANOVA with genotype as between subjects and delay as within subject factors, main effect of genotype: *F*(1,18) = 7.6, *p* < 0.05, no main effect of delay or interaction, all *F* < 1, *p* > 0.2).

Taken together, both measures of object exploration (touches and approaches) suggest that object recognition memory, even at very short delays, is impaired in TgCRND8 mice.

### 3.3 Experiment 2: visual discrimination, reversal learning, and retention

**Fig. 2:**
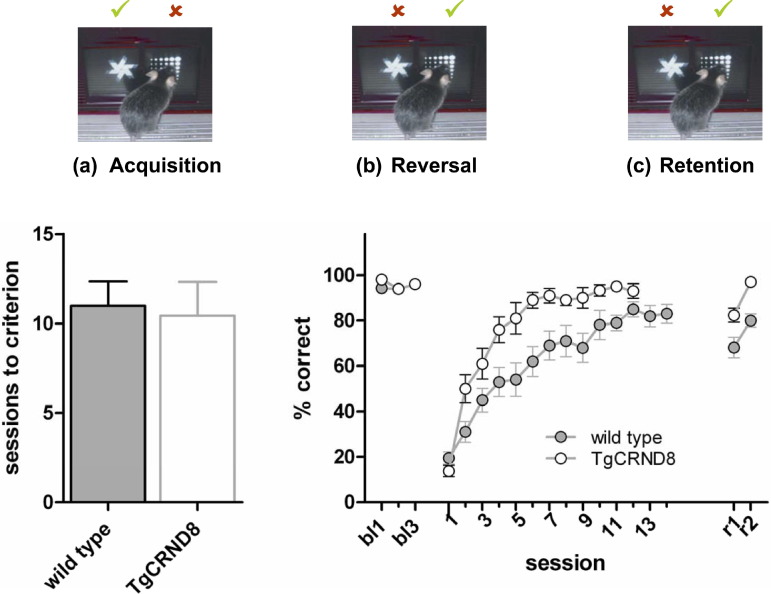
Visual discrimination, reversal, and retention. (a) Task acquisition: sessions required to reach criterion (> 80% correct responses on 2 consecutive days). (b) Performance levels (choice accuracy) during 3 sessions of baseline testing (bl1–bl3), after reversing the task contingencies (the previous S+ became S−, and the previous S− the S+), and (c) after a 10 day, test-free interval (r1 and r2). Data are presented as mean ± standard error of the mean.

A new cohort of animals (cohort 2) was tested on a visual discrimination and reversal task (4.5-month-old TgCRND8 (*n* = 7) and wild type (*n* = 7) mice). During the first phase of this task, the acquisition phase, animals learn that the touch of only 1 of 2 different stimuli on display is associated with a food reward. The acquisition phase provides information about the general ability to perceive and distinguish visual stimuli and assesses nonhippocampal, associative stimulus-reward learning. Both genotypes required a similar number of sessions to reliably choose the rewarded over the unrewarded stimulus (criterion 80% correct choices for 2 days, Fig. 2a; 1-way ANOVA, *F* < 1, *p* > 0.5). Thus, the ability to discriminate visual stimuli and nonhippocampal associative learning abilities are unaltered in TgCRND8 mice.

Following acquisition and 3 more sessions of the original discrimination task, the reward contingencies in the following sessions were reversed, i.e., the formerly rewarded stimulus was left unrewarded, whereas the formerly unrewarded stimulus was rewarded. The reversal phase (Fig. 2b) assesses cognitive flexibility, in particular the ability to inhibit previously learned associations and the capacity to relearn a new contingency for a familiar stimulus. Response accuracies on the original discrimination did not differ between genotypes, and performance after reversal initially dropped to the same level (Fig. 2b, RM ANOVA, simple main effect of genotype on session 1: *F*(1,12) = 2.4, *p* > 0.1, see details of ANOVA below). However, TgCRND8 mice subsequently acquired the reversed, new reward contingencies significantly faster than wild type mice (Fig. 2b, RM ANOVA, main effect of genotype: *F*(1,12) = 10.6, *p* < 0.01; genotype by session interaction: *F*(11,132) = 17.4, *p* < 0.001).

In order to address whether faster reversal of responding in TgCRND8 mice might be related to weaker memory of stimulus predictions, we tested the degree of retention for the current reward contingencies. After a test-free period of 10 days, TgCRND8 mice still performed above 80% correct choices (Fig. 2c), whereas wild type mice performance dropped to 68% (but note the lower baseline performance before the interval; RM ANOVA with genotype as between subjects factor, and session as within subject factor, main effect of genotype *F*(1,12) = 22.2, *p* < 0.01, no interactions involving genotype). Thus, retention of stimulus-reward associations is unaltered in TgCRND8 mice, which suggests that a weaker, shorter-lived memory is unlikely to be the reason for faster reversal learning in these animals. Rather, TgCRND8 mice more readily abandoned a previously acquired association, which suggests that pathological Aβ levels might alter cognitive flexibility.

### 3.4 Experiment 3: 5-CSRTT

Impairments of attention and impaired response control are common in patients of Alzheimer's disease (Bentley et al., 2008; Sahakian and Coull, 1993), but have not been extensively studied in mouse models of the disease. We used a new cohort of mice (cohort 3, wild type: *n* = 10; TgCRND8: *n* = 8, 4–4.5 months old at onset of testing) to assess attention and aspects of executive control with the 5-CSRTT, a task procedurally almost identical to tests of divided, sustained attention used in clinical diagnostic test batteries like Cambridge Neuropsychological Test Automated Battery (Sahakian and Coull, 1993).

#### 3.4.1 Task training

Wild type and TgCRND8 mice equally acquired the general task procedure and required a similar number of sessions to reach the criterion of stable baseline performance (> 80% correct, < 20% omissions at 2-second stimulus duration for 3 of 4 consecutive days; mean sessions to criterion, wild type mice: 11.4 ± 0.9; TgCRND8 mice: 12.3 ± 0.8; 1-way ANOVA *F* < 1, *p* > 0.1).

#### 3.4.2 Baseline performance

**Fig. 3:**
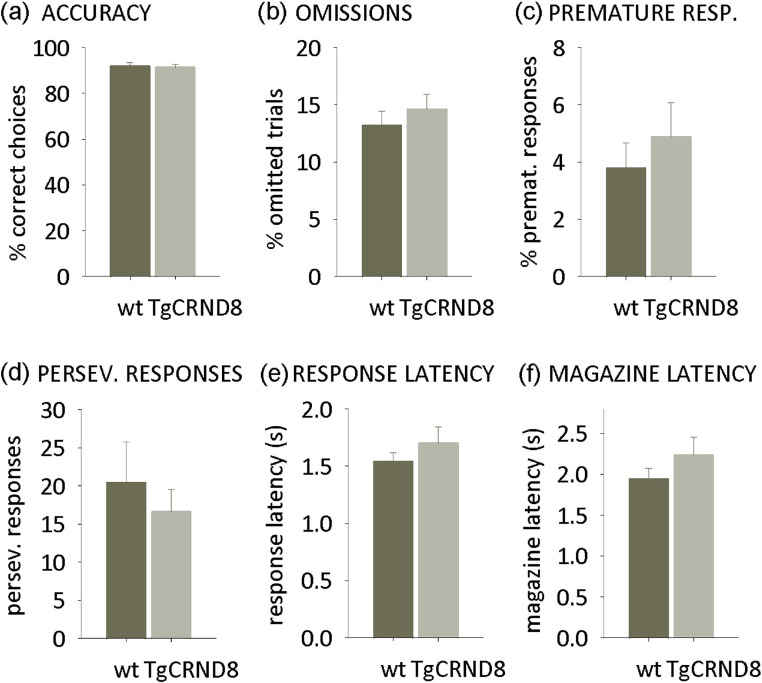
Baseline performance on the 5-choice serial reaction time task (5-CSRTT) with a 2-second stimulus duration. (a) Response accuracy excluding omitted trials. (b) Percentage of omitted trials. (c) Percentage of premature responses before appearance of the stimulus. (d) Mean total perseverative responses per session. (e) Mean latency of response. (f) Mean latency of reward collection after a correct response. Data are presented as mean ± standard error of the mean.

After reaching criterion, wild type and TgCRND8 mice performed similarly on all measures of the task (2-second stimulus duration, Fig. 3a–f). Importantly, TgCRND8 mice showed similar omission numbers (Fig. 3b), magazine, and response latencies (Fig. 3e and f) to wild type mice, which suggests that TgCRND8 performance is not compromised by gross motor or motivational abnormalities (1-way ANOVAs, all *F* < 1, *p* > 0.1).

#### 3.4.3 Probe trials

**Fig. 4:**
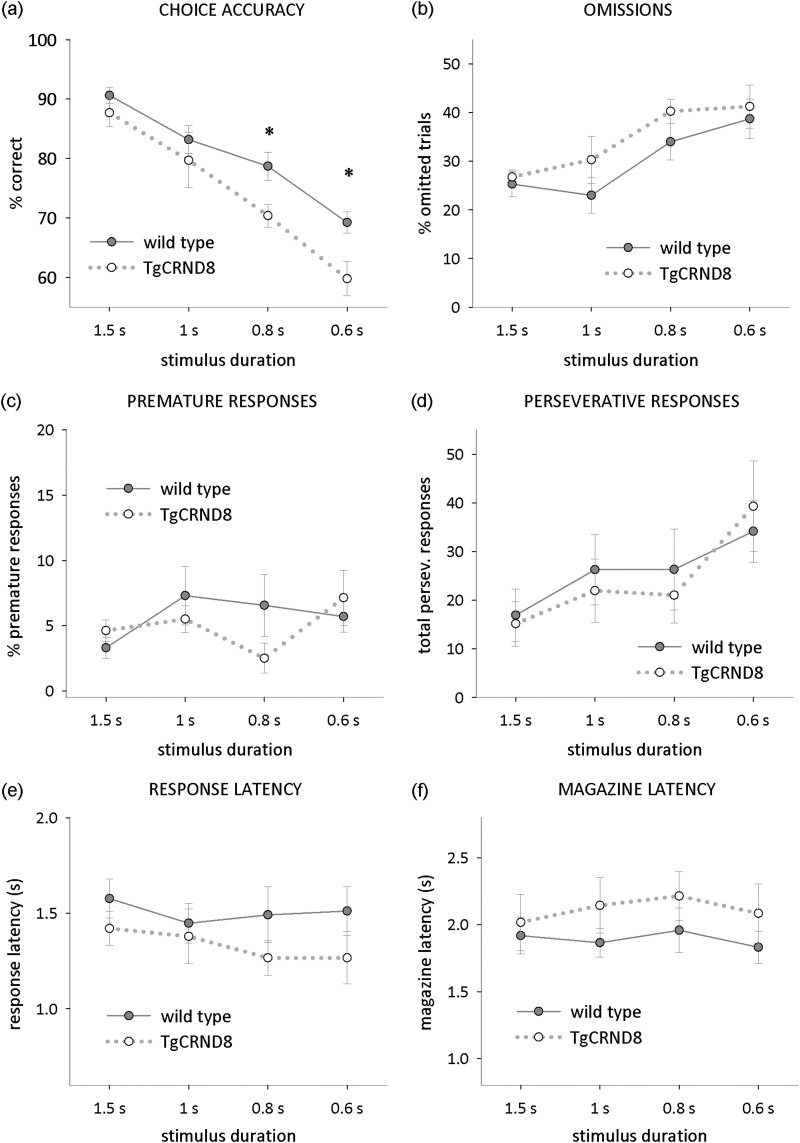
Five-choice serial reaction time task (5-CSRTT) performance on probe trials with 1.5-, 1-, 0.8-, and 0.6-second stimulus duration. (a) Response accuracy excluding omitted trials. (b) Percentage of omitted trials. (c) Percentage of premature responses before appearance of the stimulus. (d) Mean total perseverative responses per session. (e) Mean latency of response. (f) Mean latency of reward collection after a correct response. Data are presented as mean ± standard error of the mean. * Simple main effect, *p* < 0.05.

Next, we challenged TgCRND8 and wild type mice by shortening the time the stimulus remained on the screen. Under conditions of high attentional demand, i.e. with stimulus duration of 0.8 or 0.6 seconds, TgCRND8 mice responded significantly less accurately than their wild type littermates (Fig. 4a; RM ANOVA with genotype as between subjects factor, and stimulus duration as within subject factor; main effect of genotype: *F*(1,16) = 5.6, *p* < 0.05; genotype × stimulus duration interaction: *F*(1,16) = 5.7, *p* < 0.05). No differences between genotypes were observed in the number of omitted trials (Fig. 4b; RM ANOVA, same factors as above, no effects or interactions involving genotype, all *F* < 1, *p* > 0.5), response latencies (Fig. 4e; RM ANOVA, same factors as for accuracy, no effects or interactions involving genotype, all *F* < 1, *p* > 0.1), or magazine latencies (Fig. 4f; RM ANOVA, same factors as above, *F* < 1, *p* > 0.1) which suggests that the impairment was specific to the attentional processes required to detect the location of the target, rather than due to a general failure in stimulus perception, or reduced motivation. Reduced response accuracies in the absence of changes to response latencies or omissions are regarded as a main indicator for impairments of sustained attention: an incorrect response, in combination with unaltered response latencies, suggest that an animal has detected the flash of light, but failed to attend to the screen to identify the correct stimulus location.

Furthermore, 5-CSRTT performance of TgCRND8 mice showed no indication of impaired response control: neither the number of premature nor the number of perseverative responses—which are regarded as measures of impulsivity and compulsivity, respectively—were different from those of wild type mice (Fig. 4c and d; RM ANOVA with genotype as between subjects factor, and stimulus duration as within subject factor; no interactions, all *F* < 1, *p* > 0.1).

### 3.5 Experiment 4: extinction

**Fig. 5:**
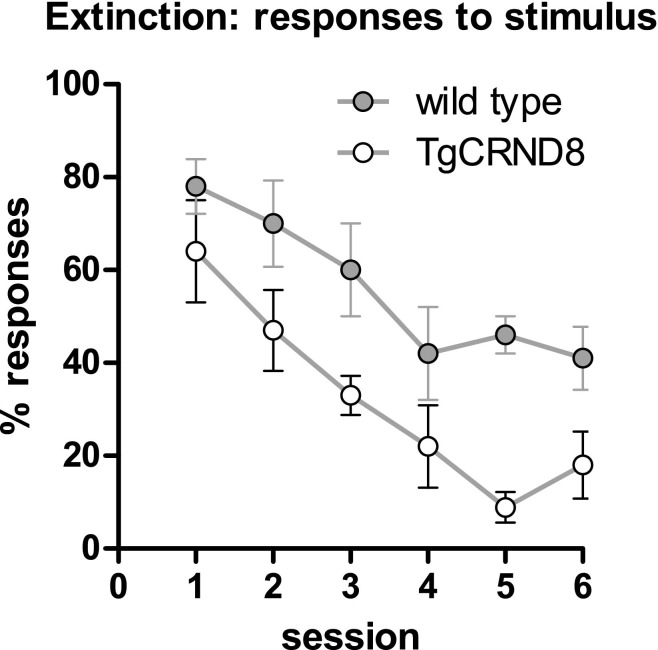
Memory extinction. Percentage of stimuli mice touched during each session (responding no longer led to a food reward). Data are presented as mean ± standard error of the mean.

Experiment 2 (reversal learning) suggested that TgCRND8 mice show altered cognitive flexibility. In order to further characterize the ability to inhibit previously acquired response-reward associations, we tested the same mice from experiment 3 (5-CSRTT, cohort 3, now 5–6 months old) on an extinction paradigm. Although there was a small possibility that the animals' previous performance on the 5-CSRTT would in some way interfere with extinction performance, the procedural and cognitive simplicity of the extinction paradigm made this very unlikely: mice were trained to respond to a single, central stimulus for a food reward. When a mouse had reached criterion, no further reward was given upon stimulus touch in the following sessions. In comparison with the reversal learning task, this test also provides an indication of how fast an animal inhibits a previously acquired response, but does not imply the relearning of new stimulus-reward associations. TgCRND8 mice perseverated less than wild type mice and stopped responding to the stimulus significantly earlier than wild type mice (Fig. 5, RM ANOVA with genotype as between subjects factor, and session as within subject factor; main effect of genotype: *F*(1,12) = 34.8, *p* < 0.001, no further effects or interactions, all *F* < 1, *p* > 0.1). In addition to the facilitated reversal learning in TgCRND8 mice (experiment 2), these findings provide evidence that TgCRND8 mice more rapidly inhibit/discard previously learned response patterns.

## 4 Discussion

### 4.1 Similar to AD patients, TgCRND8 mice have memory deficits

Impairments of memory are thought to be the most prominent impairment of preclinical AD patients as well as clinical cases (Elias et al., 2000; Small et al., 1997; Tierney et al., 1996). Spontaneous object recognition and visual discrimination learning were used to analyze different aspects of learning and memory in TgCRND8 mice. TgCRND8 mice were unimpaired on visual discrimination learning, a simple, nonspatial associative learning task that in its standard version is independent of medial temporal lobe structures (Jones and Mishkin, 1972). As previously reported for other transgenic and nontransgenic mouse strains with a C57Bl6 or 129 background (Bartko et al., 2011; Bussey et al., 2001; Morton et al., 2006; Wong and Brown, 2006), the findings provide a clear demonstration of the general ability of TgCRND8 mice (on a C57Bl/6:129sv background) to accurately perceive and discriminate 2-dimensional visual stimuli presented on a computer screen, and to associate these stimuli with the occurrence of reward.

The lack of impairment on the visual discrimination task is also consistent with findings from patients with mild cognitive impairment and early-stage AD, who are unimpaired on a similar touch screen-operated test (Lee et al., 2007).

In contrast, TgCRND8 mice were impaired on the touch screen object recognition test, which is consistent with a previously reported deficit in the standard nonautomated object recognition paradigm (Francis et al., 2012; Greco et al., 2010, Romberg et al., 2012a). However, these and our data may not be directly comparable, because the previous studies employed TgCRND8 mice on a different hybrid background (C3H:Bl6).

Spontaneous object recognition memory in rodents depends largely on a functional perirhinal cortex (Forwood et al., 2005; Winters et al., 2004). Rodents and humans with medial temporal lobe lesions, as well as AD patients, generally express delay-dependent impairments in recognition memory, sparing short-term recognition (Forwood et al., 2005; Freed et al., 1987; Parra et al., 2010; Winters et al., 2004). Unlike AD patients and subjects with medial temporal lobe lesions, however, TgCRND8 mice also performed worse than wild type mice when the delay between sample and test phase was very short (1 minute). It appears, therefore, that the touch screen version of the object recognition test may be more sensitive than the standard, nonautomated version. This could be due to the stimulus material; the objects displayed on the touch screen have fewer sensory features, in fewer sensory modalities, than real 3-D objects, possibly making them harder to remember. Consistent with this argument, rapid forgetting can be seen in nonautomated object recognition when 2-dimensional stimulus cards are used instead of 3-D objects (Forwood et al., 2007).

Taking the reduced salience of 2-dimensional stimuli into account, the present memory impairments after both short and long delays may be related to findings that object recognition memory at short delays is not necessarily independent of medial temporal lobe structures; Bartko et al. (2007) have shown that perirhinal cortex lesions in rats impair spontaneous object recognition with a minimal delay when objects are made difficult to discriminate.

Interestingly, wild type mice also had lower discrimination scores on the short delay compared with the long delay version of the task (although this difference was not statistically evident), which suggests that the short delay version might be more demanding. Although one would intuitively expect memory to be better at shorter delays, the short delay paradigm may be more difficult because the familiar object retains some degree of novelty when there is such a short delay between the sample and test phases, or because of advanced proactive interference from the sample phase (Romberg et al., 2012a).

Regardless of delay, object recognition memory appears to be impaired in TgCRND8 mice, which may be related to abundant AD-typical Aβ pathology in structures known to be critical to this function, such as the perirhinal cortex and the hippocampus (Adalbert et al., 2009; Chishti et al., 2001).

### 4.2 Behavioral flexibility is altered in TgCRND8 mice

Although unimpaired on the acquisition of a visual discrimination task, TgCRND8 mice performed differently from wild type mice when reward contingencies were reversed. In addition to measuring the ability to form further, new stimulus-reward associations, the reversal test provides an indication of behavioral flexibility and the ability to inhibit previously rewarded behavior (Bussey et al., 1997; Jones and Mishkin, 1972). TgCRND8 mice reversed responding more rapidly than wild type mice. At least 2 neuropsychological mechanisms could potentially account for such behavior: TgCRND8 mice might have formed a weaker original stimulus-reward association, or they might be able more readily to inhibit prepotent responses to previously correct stimuli. However, we also showed that long-term retention, i.e., the ability to remember previously acquired information after a long delay (10 days), was unaltered in TgCRND8 mice, which provides some evidence against weaker stimulus-reward associations in TgCRND8 mice. Furthermore, TgCRND8 mice were also faster than wild type mice to extinguish responding in a simple extinction test, which does not require the learning of new stimulus-reward associations.

Interestingly, a few other studies have found evidence for increased response inhibition in different mouse models of AD. Mice overexpressing p25 show a facilitation of spatial reversal learning, and accelerated extinction of fear memory (Angelo et al., 2003; Sananbenesi et al., 2007). P25 is generated by cleavage of the cyclin-dependent kinase 5 activator p35 and accumulates in the forebrain of AD patients (Angelo et al., 2003). Cyclin-dependent kinase 5 in turn hyperphosphorylates tau, a mechanism downstream of Aβ-accumulation thought to be a major cause of neuritic dystrophy. In addition, more rapid extinction, albeit of previously acquired taste aversion, was also found in P301L tau transgenic mice (Pennanen et al., 2004).

Thus, TgCRND8 mice and other mouse models of AD may have a greater tendency to discard or inhibit previously acquired response rules. However, it is important to consider that facilitated reversal learning and/or extinction represent abnormal behavior that may not necessarily be beneficial in all situations, and could contribute to impairments on other cognitive tasks.

Alterations of cognitive flexibility are often associated with a dysfunction of the prefrontal cortex, one of the brain regions heavily affected by AD-typical pathology in patients and TgCRND8 mice (Adalbert et al., 2009; Braak and Braak, 1991; Chishti et al., 2001). However, lesions of the orbitofrontal cortex (Chudasama and Robbins, 2003; Jones and Mishkin, 1972) increase perseverative responding to the previously rewarded stimulus and thus impair reversal learning. The finding of faster reversal learning in the CRND8 mice suggests that any pathology present in the orbitofrontal cortex was not sufficient to cause impairments in reversal learning. However it has been reported that medial prefrontal cortex lesions cause faster reversal learning in mice, suggesting that pathology in this region of prefrontal cortex may have contributed to the pattern of results seen here (Graybeal et al., 2011).

Little work has been done studying reversal learning and extinction in AD patients, probably because behavioral flexibility is thought to be largely intact until the late stages of the disease (Sahakian et al., 1990). While we also found no evidence that reversal learning and extinction are slower in TgCRND8 mice, the faster reversing and extinction we observed in these mice may indicate an interesting construct worth investigating further in humans and animal models.

### 4.3 TgCRND8 mice show deficits in sustained attention

We found that TgCRND8 mice responded less accurately than wild type mice to short, spatially unpredictable stimuli, which is commonly interpreted as a deficit in sustained attention (Robbins, 2002). Importantly, TgCRND8 mice did not omit more trials than wild type mice, which suggests that neither motivational nor motor abnormalities, nor a gross failure to attend to the stimulus display area, contributed to poor accuracy on the task. Unaltered magazine and response latencies, as well as unchanged gross activity and exploration levels (assessed during the sample phase of the spontaneous object recognition paradigm) also argue against such an explanation. Although it is possible that the reported changes in accuracy reflect effects on basic visual sensory function, we think this is unlikely, because TgCRND8 mice responded as accurately as wild type mice at longer stimulus durations, and were unimpaired on visual discrimination tests.

The choice accuracy deficit of TgCRND8 mice was comparable with a similar deficit we previously reported in the 3xTgAD mouse model of Alzheimer's disease (Romberg et al., 2011, Romberg et al., 2012b). Because 3xTgAD mice harbor 3 major AD-related mutations in the tau, presenilin and APP genes, whereas TgCRND8 mice only carry the APP_swe_-transgene, our present findings suggest that the sustained attention deficits in both AD mouse models may be attributed to the APP mutation rather than the tau mutation. In contrast, we found that unlike 3xTgAD mice, TgCRND8 mice showed no alterations in perseverative responding. Thus, increased perseverative responses previously described in 3xTgAD mice may relate to the additional tau P301L transgene.

In rats, selective response accuracy changes on the 5-CSRTT correlate significantly with cholinergic cell loss in the nucleus basalis of Meynert, and with reductions in acetylcholine efflux in the medial prefrontal cortex (Dalley et al., 2004; McGaughy et al., 2002). Thus, the selective accuracy decrement in TgCRND8 mice may reflect a deficit in cholinergic signaling (or other pathological changes) in the medial prefrontal cortex, an area heavily affected by Aβ deposits in TgCRND8 mice (Adalbert et al., 2009; Chishti et al., 2001).

Sustained attention deficits similar to those we describe here for TgCRND8 mice are well documented in human mild cognitive impairment and AD patients (Baddeley et al., 2001; Bentley et al., 2003, 2008; Berardi et al., 2005; Foldi et al., 2005; Grady et al., 1988; Perry and Hodges, 1999; Perry et al., 2000; Reid et al., 1996; Sahakian and Coull, 1993; Sahakian et al., 1989, Romberg et al., 2012b). Specifically, Sahakian and Coull (1993) found response accuracy decrements in mild cognitive impairment patients on a touch screen-operated choice reaction time test from the Cambridge Neuropsychological Test Automated Battery which is analogous to the 5-CSRTT we used here. Given that attention deficits are likely to impact on tests of memory—if a stimulus is not well-attended it may not be well-encoded—it is conceivable that the attentional deficit of TgCRND8 mice might have contributed to some of the memory impairment in the object recognition test described above.

### 4.4 Conclusions

The present findings demonstrate how a fully automated touch screen battery can be used to characterize the cognitive abilities of a typical mouse model of AD. In contrast to previous approaches that have assessed either memory (e.g., Francis et al., 2012) or attention (Romberg et al., 2011) in transgenic AD mouse models, a major advantage of the present study is that both of these functions, as well as cognitive flexibility and associative learning, were assessed simultaneously, using tasks with similar sensory-motor, procedural demands.

Similar to AD patients, TgCRND8 mice were unimpaired on simple association-memory tasks independent of the medial temporal lobe, and showed profound deficits on the spontaneous object recognition test. Moreover, these tests revealed that TgCRND8 mice, like early AD patients, have deficits in sustained attention. Our results also suggest that behavioral flexibility is altered in TgCRND8 mice, a finding that may require further investigation in mice and humans. While the described memory deficits in this mouse model of AD are well established and confirm the validity of our touch screen-operated object recognition memory task, alterations in attention and response control have rarely been assessed in mouse models of AD. However, the described attention and response control deficits/facilitations may be of particular interest and importance, because they are likely to influence performance on memory tasks, tests widely used to assess the efficacy of pharmacological treatment.

## Disclosure statement

L.M.S. and T.J.B. consult for Campden Instruments. The authors disclose no conflicts of interest.

All experimentation was conducted in accordance with the United Kingdom Animals (Scientific Procedures) Act (1986).

## Figures and Tables

**Table 1:** Automated touch screen-operated cognitive tests used in this study

Touch screen task	Cognitive function measured	Important brain structures	Human AD evidence
Visual discrimination	Visual perception, basic nonhippocampal associative learning	Visual areas, striatum	Not affected
Spontaneous object recognition	Memory	Medial temporal lobe structures especially perirhinal cortex	Affected
Five-choice serial reaction time task	Attention, response control	Prefrontal and parietal cortex, cholinergic projections	Attention affected, response control impaired at later stages
Reversal learning	Cognitive flexibility	Prefrontal cortex	Affected only at later stages
Extinction	Response Inhibition	Various structures depending on paradigm, including prefrontal cortex, hippocampus	Affected only at later stages

Key: AD, Alzheimer's disease.
